# Interferon-γ release assay and mantoux response in infants with tuberculous meningitis in low and intermediate burden countries

**DOI:** 10.1186/s12879-023-08327-4

**Published:** 2023-05-30

**Authors:** Yan-Hua Yang, Jie Hou, Yu He, Yan-An Zhang, Mao-Shui Wang

**Affiliations:** 1 The Immunetech Institute of Guilin, Guilin, 541004 China; 2 Department of Intensive Care, Affiliated Hospital of Shandong University of Traditional Chinese Medicine, Jinan, 250011 China; 3 Department of Clinical Laboratory, First Affiliated Hospital of Guangxi Medical University, Nanning, 530012 China; 4 Department of Cardiovascular Surgery, Shandong Public Health Clinical Center, Shandong University, 46# Lishan Road, Jinan, 250013 PR China; 5 Shandong Key Laboratory of Infectious Respiratory Disease, Jinan, Shandong China; 6 Department of Lab Medicine, Shandong Public Health Clinical Center, Shandong University, 46# Lishan Road, Jinan, 250013 PR China

**Keywords:** Infant, Tuberculous meningitis, Interferon-γ release assay, Mantoux test, Diagnosis

## Abstract

**Aim:**

Until now, the performance of interferon-γ release assay (IGRA) and Mantoux tests remains unclear in infant tuberculous meningitis (TBM). Therefore, a systematic review is performed to evaluate the sensitivity of IGRA and Mantoux tests for the diagnosis of infant TBM in low and intermediate tuberculosis (TB) burden countries, while following PRISMA.

**Methods:**

Several databases, including PubMed, EBSCO, Embase, Scopus, Web of Science, ClinicalTrials.gov, and Cochrane Central Register of Controlled Trials, were searched. Articles describing the results of IGRA or Mantoux tests among infant TBM were included for analysis. Data, such as age, sex, Mantoux test or IGRA, and cerebrospinal fluid (CSF) microbiological examinations (such as acid-fast bacilli (AFB) smear, TB PCR, and TB culture), were extracted from each study.

**Results:**

A total of 31 articles were enrolled for further analysis, including 48 cases. The mean age was 9.4 ± 5.8 months and boys accounted for 57.1% of infants (24/42). Mantoux test was positive in 57.4% (27/47) of tested infants and IGRA was positive in 77.8% (7/9) of infants. In addition, among the infants with confirmed TB, 18 (52.9%, 18/34) of them have positive Mantoux responses and 7 (20.0%, 7/35) have positive IGRA results.

**Conclusions:**

In low or intermediate TB burden countries, the Mantoux test has a poor performance for diagnosing TBM among infants, and IGRAs appear to have a moderate sensitivity for the diagnosis of infant TBM.

**Supplementary Information:**

The online version contains supplementary material available at 10.1186/s12879-023-08327-4.

## Background

Currently, tuberculosis (TB) remains a major threat to child health. According to the WHO Global Tuberculosis Report (2021), 9.9 million people were estimated to fall ill with TB and children accounted for 11% of them [1]. As a severe form of TB, tuberculous meningitis (TBM) accounted for approximately 10% of all TB children [2]. Moreover, a meta-analysis revealed a death rate of 19.3% in TBM children [3]. Fortunately, accumulated evidence is made in terms of the management of childhood TBM, and a declined TBM incidence was observed in a cross-sectional study from 2006 to 2011 [4]. However, the diagnosis of childhood TBM is often a challenge because of the nonspecific clinical presentation and the lack of a sufficiently sensitive microbiological tool for the diagnosis. Therefore, further investigation is required to improve the strategy of diagnosis for this disease,

Young age is considered a potent risk factor for TBM, while Bacillus Calmette-Guérin (BCG) vaccination is protective, particularly in young children [5]. Infant TBM is rarely reported. However, In China, TBM accounted for 19.1% of all TB infants [6]. Currently, almost all studies of infant TBM are case reports and no reliable evidence is provided to improve its management, and most treatment choices are copied from the experience of childhood TBM directly. Hence, an effort is needed to review the characteristics of infant TBM and improve its current dilemma.

In general, the diagnostic value of interferon-γ release assay (IGRA) or Mantoux response is limited in high TB burden countries, and usually no intervention is given when there is a positive result. However, in low and intermediate TB burden countries, if positive results for interferon-γ release assay (IGRA) or Mantoux response were observed, isoniazid preventive therapy may be then initiated after active TB is ruled out, and the value of positive results were therefore significant. Besides, these tests could facilitate the diagnosis of TBM [7]. Unfortunately, false-negative results of IGRA remain a concern among childhood TB, and approximately 15% of culture-confirmed TB children were IGRA-negative [8], and children with an age of < 2 years were more likely to have a negative IGRA response [9]. Therefore, the actual performance of IGRA and Mantoux tests require to be investigated, especially in infant TB. In this study, a systematic review is performed aiming to evaluate the sensitivity of IGRA and Mantoux tests for diagnosing infant TBM.

## Methods

### Literature searching

On October 14th, 2021, several databases, including PubMed, EBSCO, Embase, Scopus, Web of Science, ClinicalTrials.gov, and Cochrane Central Register of Controlled Trials, were searched, while following PRISMA guidelines. The full search strategies are described in Supplementary materials. Two authors independently screened the reports, and a third arbitrated disagreements between them (HJ, HY, and WMS).

### Eligibility criteria

Articles describing the results of IGRA or Mantoux tests among infant TBM were eligible for inclusion. Infants were defined as ≤ 24 months. There is no requirement of study design or language. Duplicates were automatically detected by a reference manager and reduced to a single one. Other exclusion criteria included: published before 2000, unavailable full text, Non-TBM, no IGRA or Mantoux data, and duplicates. Data extracted from each study included the following items: first author, country, age, sex, Mantoux test or IGRA, cerebrospinal fluid (CSF) microbiological examinations (such as acid-fast bacilli (AFB) smear, TB PCR, and TB culture), *Mycobacterium tuberculosis*, Bacille Calmette-Guérin (BCG) vaccination. A diagnosis of confirmed TB (or TBM) was made if non-central nervous system (non-CNS, or CNS) samples were AFB smear-, TB-PCR- or culture-positive.

### Statistical analysis

Data were analyzed using SPSS 16.0. Continuous variables were reported as median ± interquartile range (IQR) and categorical variables were reported as frequencies (percentages). Continuous variables were also transformed into categorical variables for further analysis if applicable. In addition, the association between Mantoux results and age or sex was analyzed using univariate logistic regression analysis.

## Results

### Literature selection

**Fig. 1:**
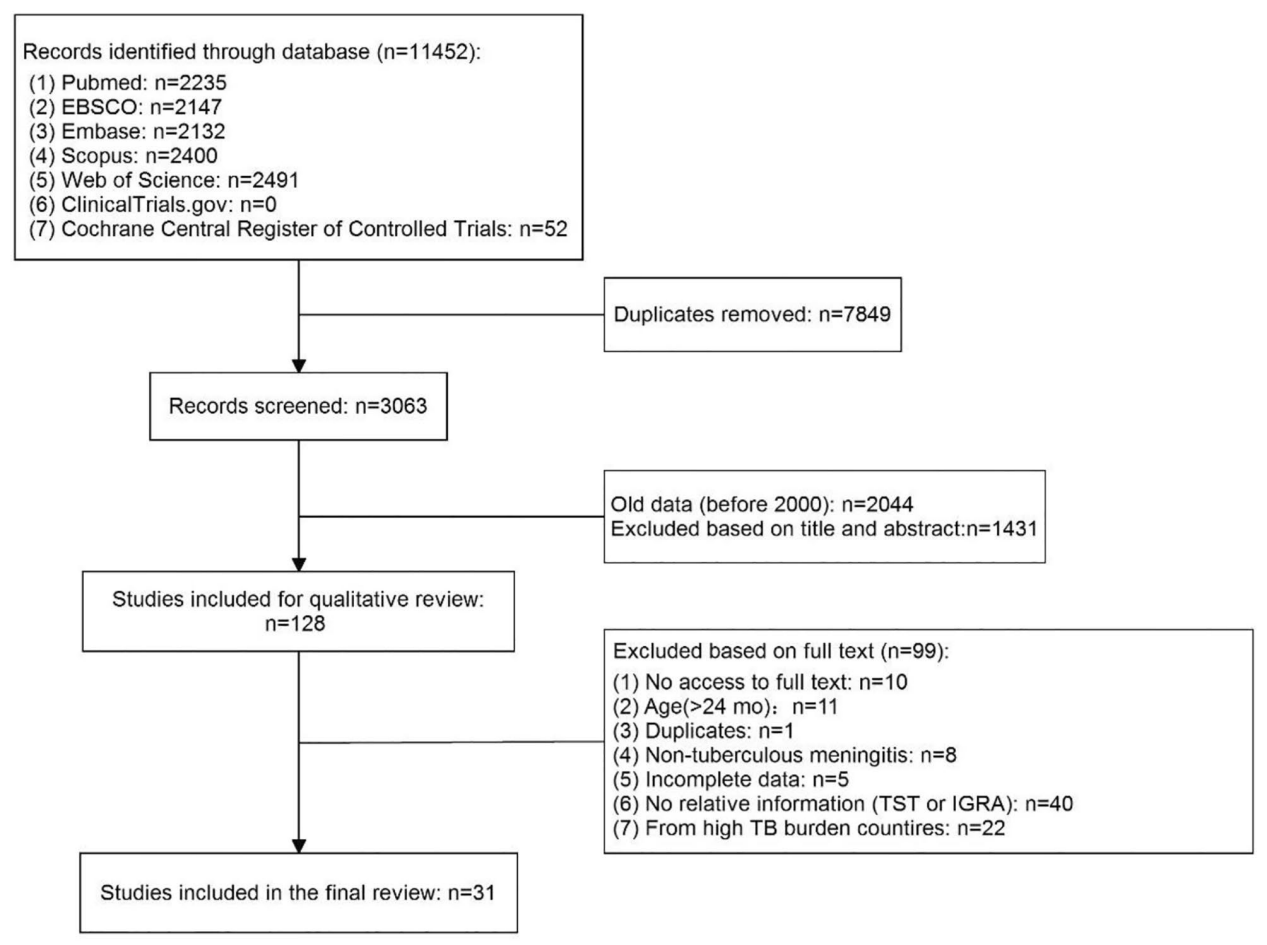
Literature selection

A total of 11,452 records were identified, and then duplicates (n = 7849) were removed. The remaining records (n = 3063) were screened and excluded based on title and abstract, and only 128 articles were left for qualitative review. Subsequently, 99 articles were excluded due to: unavailable full text (n = 10); age (> 24 months; n = 11); duplicates (n = 1); non-TBM (n = 8); incomplete data (n = 5), no TST or IGRA results (n = 40), and high TB burden countries (n = 22). Finally, 31 articles were enrolled for further analysis, including 48 cases (Fig. 1).

### Baseline characteristics

The mean age was 9.4 ± 5.8 months and boys accounted for 57.1% of infants (24/42). Mantoux test was positive in 57.4% (27/47) of tested infants and IGRA was positive in 77.8% (7/9) of infants. Of the 47 cases, twenty-six were confirmed with CSF findings, including AFB smear (+, n = 4), PCR (+, n = 13), and culture (+, n = 18). Non-CNS TB evidence were found in 27 patients, including AFB (+, n = 10), PCR (+, n = 9), and culture (+, n = 20). Based on non-CSF and CSF findings, a total of 35 cases were found with microbiological evidence. The details are listed in Supplementary Table 1.

### Mantoux and IGRA results

The Mantoux and IGRA results are listed in Table 1. Of the infants with confirmed TBM (n = 26), 25 (96.2%, 25/26) were tested with Mantoux test and 14 (56.0%, 14/25) of them were positive, 6 (23.1%, 6/26) were tested with IGRAs and 4 (66.7%, 4/6) of them were positive. Of the infants with confirmed TB elsewhere (non-CNS, n = 27), 27 (100%, 27/27) were tested with Mantoux test and 17 (63.0%, 17/27) of them were positive, 5 (18.5%, 5/27) were tested with IGRAs and 4 (80.0%, 4/5) of them were positive. In addition, among the infants with confirmed TB (n = 35), 34 (97.1%, 34/35) were tested with Mantoux test and 18 (52.9%, 18/34) of them were positive, 7 (20.0%, 7/35) were tested with IGRAs and 5 (71.4%, 5/7) of them were positive. IGRAs and Mantoux test were performed in 9 infants, discordance between IGRAs and Mantoux results was found in three cases (including an indeterminate response, n = 1).

**Table 1:** The Mantoux and IGRA results of TBM infants

		Total	Mantoux results (n)		IGRA (n)	
			Negative (< 5 mm)	Positive (≥ 5 mm)	Negative, or Indetermine	Positive
Number		48	20	27	2	7
Age (months)		9.4 ± 5.8	8.4 ± 5.9	9.9 ± 5.8	17.0 ± 1.4	12.7 ± 5.4
Sex (male, %)		57.1% (24/42)	42.1% (8/19)	43.5% (10/23)	100% (2/2)	42.9% (3/7)
Microbiological TB evidence						
	CNS (-)	22	9	13	0	3
	CNS (+)	26	11	14	2	4
	Non-CNS (-)	21	10	10	1	3
	Non-CNS (+)	27	10	17	1	4
	Total (-)	13	4	9	0	2
	Total (+)	35	16	18	2	5
BCG vaccination (n = 36)						
	Yes	16	7	8	1	0
	No	20	8	12	1	6
*M.TB* (n = 33)						
	Yes	32	13	19	1	6
	No, BCG	1	0	0	1	0

IGRA, Interferon-γ Release Assay; TBM, tuberculous meningitis; TB, tuberculosis; CNS, central nervous system; BCG, Bacille Calmette-Guérin; M.TB, Mycobacterium Tuberculosis

In addition, among infants with BCG vaccination (n = 15), 8 (53.3%) of them were Mantoux-positive. An interesting finding was also observed, this is that three TBM infants have conversion results of Mantoux test (before anti-TB therapy, n = 1; during anti-TB therapy, n = 2). Further analysis was performed to evaluate the association between age (or sex) and Mantoux responses. However, no significant correlation was found (age, P = 0.405; sex, P = 0.829).

## Discussion

Infants are considered at high risk of TBM. First, infants have a higher likelihood of progression from infection to active TB and even dissemination occurs, leading to severe TB disease (such as TBM) [10]. Second, infants have underdeveloped cell-mediated immunity, which could lead to a rapid progress of the disease before the initiation of treatment. Third, due to the anatomical underdevelopment of cranial arteries and narrow cerebrospinal passages, cerebral infarction and hydrocephalus occur easily, following meningeal changes caused by immunological response against TB. Unfortunately, the tools for diagnosis of infant TBM are imperfect and incompletely characterized, this disadvantage makes the management more difficult.

As known, most studies of TBM infants were described as case reports. To our knowledge, the study is the first report to investigate the diagnostic yield of IGRA or Mantoux test for infant TBM. This systematic review demonstrated that in low and intermediate burden countries, TBM infants have a relatively low positive rate of Mantoux test and a relatively high positivity of IGRA. Similarly, a low positive rate of Mantoux response was observed in TBM infants with BCG vaccination. Therefore, caution is required to understand the value of Mantoux responses among these countries. As known, a false-negative Mantoux response would lower the suspicion of TB disease significantly.

In the study, among the infants with confirmed TB, approximately half of them have a positive Mantoux response. Moreover, previous BCG immunization had no significant effect on the reaction to tuberculin and repeat Mantoux test may increase its positive rate. In a meta-analysis, it was demonstrated that Mantoux test has a comparable sensitivity with IGRAs (ELISA, 70%; ELISPOT, 62%) for the diagnosis of childhood TB [11], the poor performance of Mantoux test remains a significant concern for childhood TB, especially in infants. Duque-Silva A, et al. described the epidemiology of pediatric CNS TB in USA, and found that Mantoux test results were negative in 38.2% of 170 CNS TB cases tested [12]. Furthermore, Mantoux test was significantly more likely to be negative in children with younger age [13]. In Papua New Guinea, Murtagh K found that among confirmed children under 2 years, 25 (71%) showed no Mantoux reaction [14]. Besides, in children with younger age, the discordance between TST and IGRA are more common than the other age group [15]. We believe that the Mantoux test has limited contribution for infant TBM diagnosis in low TB burden counties. However, based on our findings, a repeat Mantoux test is highly recommended, this would facilitate the diagnosis of infant TB. Because, this point has not been recommended for the diagnosis of infant TB.

IGRAs showed a good performance for infant TBM diagnosis, with a positive rate of 77.8%. The usefulness of IGRA in infant TB has been proved previously. For example, QuantiFERON-TB Gold In-Tube test was evaluated in 19 French immunocompetent children with active TB, and the rates of positivity were 6/10 and 9/9 in < 2 and 2- to 5-year-old children, respectively [16]. Similar findings were observed in another study: Compared with culture, the sensitivity of QuantiFERON-TB-Gold in-tube assay was 93.9%, 100%, and 94.4% in TB children ≤ 2, 2 to 5, and 5 to 16 years of age, respectively [17]. However, according to the guidelines published by the CDC, caution is warranted when using IGRAs in children aged < 5 years [18]. This is because few performance data exist for IGRA in children with younger age. Although IGRAs appear to be a good diagnostic tool for infant TBM, several cautions should be paid, when using the IGRA for infant TBM diagnosis. First, the false-negative result in confirmed childhood TB remains a concern [8]; second, TBM is known as a risk factor for false-negative IGRA results [19, 20]; third, children with younger age were significantly associated with indeterminate or negative IGRA results [21, 22].

Although our study provides valuable insights concerning infant TBM, several limitations must be recognized. First, this study has a retrospective nature, a selection bias is unavailable. Second, the sample size is small, data must be updated if new studies are published, especially IGRAs results. Third, due to the data must be collected from previous reports, the data-missing problem should be acknowledged, such as the measurement value of Mantoux response. Fourth, in our study, a 5 mm cut-off value was used for Mantoux response. Hence, the positive rate of Mantoux test is over-determined, especially in infants with BCG vaccination [23]. Additionally, since only TBM patients were included, the specificity has not been evaluated.

## Conclusions

In low or intermediate TB burden countries, the Mantoux test has a poor performance for diagnosing TBM among infants. However, a repeat Mantoux test may improve its performance. Moreover, IGRAs appear to be an accurate tool for infant TBM diagnosis, although further evidence is required to validate it.

## Electronic supplementary material

Below is the link to the electronic supplementary material.

Supplementary Table 1. The characteristics of included cases.

## Data Availability

The data used to support the findings of this study are included within the supplementary information file(s).
