# Supplementary material for: Interferon-γ release assay and mantoux response in infants with tuberculous meningitis in low and intermediate burden countries
Source: BMC Infect Dis. 2023 May 30;23:364. doi: 10.1186/s12879-023-08327-4 (PMC10230717; doi:10.1186/s12879-023-08327-4)
Supplement: Supplementary file 1 — Supplementary Table 1. The characteristics of included cases. [file 12879_2023_8327_MOESM1_ESM.docx]

**Electronic search strategy**

All search run: October 14, 2021

**1.** **PubMed**

(((“Mycobacterium tuberculosis” OR tuberculosis) and (“Cerebrospinal fluid” OR CSF OR Meningeal)) OR “Tuberculous meningitis” OR TBM OR “Tuberculosis, Meningeal”) and (Infancy OR infant OR infan* OR neonate OR neonat* OR baby OR babies OR fetus OR fetal OR foet* OR toddler OR “new born” OR “new born*” OR newborn OR newborn*)

**2.EBSCO**

| S9 | S7 AND S8 |
| --- | --- |
| S8 | Infancy OR infant OR infan* OR neonate OR neonat* OR baby OR babies OR fetus OR fetal OR foet* OR toddler OR “new born” OR “new born*” OR newborn OR newborn* |
| S7 | S3 OR S4 OR S5 OR S6 |
| S6 | “Tuberculosis, Meningeal” |
| S5 | TBM |
| S4 | “Tuberculous meningitis” |
| S3 | S1 AND S2 |
| S2 | “Cerebrospinal fluid” OR CSF OR Meningeal |
| S1 | “Mycobacterium tuberculosis” OR tuberculosis |

**3.Embase**

| #29 | # 12 AND #28 |
| --- | --- |
| #28 | #13 OR #14 OR #15 OR #16 OR #17 OR #18 OR #19 OR #20 OR #21 OR #22 OR #23 OR #24 OR #25 OR #26 OR #27 |
| #27 | newborn* |
| #26 | newborn |
| #25 | ‘new born*’ |
| #24 | ‘new born’ |
| #23 | toddler |
| #22 | foet* |
| #21 | fetal |
| #20 | fetus |
| #19 | babies |
| #18 | baby |
| #17 | neonat* |
| #16 | neonate |
| #15 | infan* |
| #14 | infant |
| #13 | Infancy |
| #12 | #8 OR #9 OR #10 OR #11 |
| #11 | ‘Tuberculosis, Meningeal’ |
| #10 | TBM |
| #9 | ‘Tuberculous meningitis’ |
| #8 | #3 AND #7 |
| #7 | #4 OR #5 OR #6 |
| #6 | Meningeal |
| #5 | CSF |
| #4 | Cerebrospinal fluid |
| #3 | #1 OR #2 |
| #2 | tuberculosis |
| #1 | “Mycobacterium tuberculosis” |

**4. Scopus**

## ( ( ( TITLE-ABS-KEY ( "Mycobacterium tuberculosis" ) OR TITLE-ABS-KEY ( tuberculosis ) ) AND ( TITLE-ABS-KEY ( "cerebrospinal fluid" ) OR TITLE-ABS-KEY ( csf ) OR TITLE-ABS-KEY ( meningeal ) ) ) OR TITLE-ABS-KEY ( "Tuberculous meningitis" ) OR TITLE-ABS-KEY ( tbm ) OR TITLE-ABS-KEY ( tuberculosis, AND meningeal ) ) AND ( TITLE-ABS-KEY ( infancy ) OR TITLE-ABS-KEY ( infant ) OR TITLE-ABS-KEY ( infan* ) OR TITLE-ABS-KEY ( neonate ) OR TITLE-ABS-KEY ( neonat* ) OR TITLE-ABS-KEY ( baby ) OR TITLE-ABS-KEY ( babies ) OR TITLE-ABS-KEY ( fetus ) OR TITLE-ABS-KEY ( fetal ) OR TITLE-ABS-KEY ( foet* ) OR TITLE-ABS-KEY ( toddler ) OR TITLE-ABS-KEY ( "new born" ) OR TITLE-ABS-KEY ( "new born*" ) OR TITLE-ABS-KEY ( newborn ) OR TITLE-ABS-KEY ( newborn* ) )

**5. Web of Science**

| #7 | #5 AND #6 Databases= WOS, BIOSIS, CSCD, DRCI, DIIDW, KJD, MEDLINE, RSCI, SCIELO Timespan=All years Search language=Auto |
| --- | --- |
| #6 | TOPIC: (Infancy) OR TOPIC: (infant) OR TOPIC: (infan*) OR TOPIC: (neonate) OR TOPIC: (neonat*) OR TOPIC: (baby) OR TOPIC: (babies) OR TOPIC: (fetus) OR TOPIC: (fetal) OR TOPIC: (foet*) OR TOPIC: (toddler) OR TOPIC: (“new born”) OR TOPIC: (“new born*”) OR TOPIC: (newborn) OR TOPIC: (newborn*) Databases= WOS, BIOSIS, CSCD, DRCI, DIIDW, KJD, MEDLINE, RSCI, SCIELO Timespan=All years Search language=Auto |
| #5 | #3 OR #4 Databases= WOS, BIOSIS, CSCD, DRCI, DIIDW, KJD, MEDLINE, RSCI, SCIELO Timespan=All years Search language=Auto |
| #4 | TOPIC: (Tuberculous meningitis) OR TOPIC: (TBM) OR TOPIC: (Tuberculosis, Meningeal) Databases= WOS, BIOSIS, CSCD, DRCI, DIIDW, KJD, MEDLINE, RSCI, SCIELO Timespan=All years Search language=Auto |
| #3 | #1 AND #2 Databases= WOS, BIOSIS, CSCD, DRCI, DIIDW, KJD, MEDLINE, RSCI, SCIELO Timespan=All years Search language=Auto |
| #2 | TOPIC: (Mycobacterium tuberculosis) OR TOPIC: (tuberculosis) Databases= WOS, BIOSIS, CSCD, DRCI, DIIDW, KJD, MEDLINE, RSCI, SCIELO Timespan=All years Search language=Auto |
| #1 | TOPIC: (Cerebrospinal fluid) OR TOPIC: (CSF) OR TOPIC: (Meningeal) Databases= WOS, BIOSIS, CSCD, DRCI, DIIDW, KJD, MEDLINE, RSCI, SCIELO Timespan=All years Search language=Auto |

**6. ClinicalTrials.gov**

(((“Mycobacterium tuberculosis” OR tuberculosis) and (“Cerebrospinal fluid” OR CSF OR Meningeal)) OR “Tuberculous meningitis” OR TBM OR “Tuberculosis, Meningeal”) and (Infancy OR infant OR infan* OR neonate OR neonat* OR baby OR babies OR fetus OR fetal OR foet* OR toddler OR “new born” OR “new born*” OR newborn OR newborn*)

**7. Cochrane Central Register of Controlled Trials (CENTRAL)**

Cochrane Controlled Register of Trials electronic databases

(((“Mycobacterium tuberculosis” OR tuberculosis) and (“Cerebrospinal fluid” OR CSF OR Meningeal)) OR “Tuberculous meningitis” OR TBM OR “Tuberculosis, Meningeal”) and (Infancy OR infant OR infan* OR neonate OR neonat* OR baby OR babies OR fetus OR fetal OR foet* OR toddler OR “new born” OR “new born*” OR newborn OR newborn*)

Supplementary Table 1. The characteristics of included cases.

| Authors | Country | Age (Months) | Sex | AFB | PCR | Culture | Microbiological TB evidence | | BCG vaccination | Mantoux results (mm or +/-) | IGRA | *M.TB* |
| --- | --- | --- | --- | --- | --- | --- | --- | --- | --- | --- | --- | --- |
|  |  |  |  |  |  |  | CNS | Non-CNS |  |  |  |  |
| Janner D, et al. [1] | USA | 8 | Male |  |  | Gastric aspirates (+); Bronchoscopy specimens (+), CSF (-) | N | Y |  | 15 |  | Y |
| Pejham S, et al. [2] | USA | 2 | Female | Ear discharge (-); CSF (-); Gastric aspirates (-); Cervical lymph node and left mastoid bone biopsy: caseating granulomas, AFB (+) |  | CSF (+); Mastoid tissue (+); Gastric aspirates (+); Middle ear fluid (+) | Y | Y |  | 12 |  | Y |
| Tung Y R, et al. [3] | Taiwan | 5 | Male/unclear | CSF (-) |  | CSF (-) | N | N | Y | (+) |  |  |
| Tung Y R, et al. [3] | Taiwan | 10 | Male/unclear | CSF (-) |  | CSF (-) | N | N | Y | (+) |  |  |
| Tung Y R, et al. [3] | Taiwan | 8 | Male | CSF (-) |  | CSF (-) | N | N | Y | (+) |  |  |
| Tung Y R, et al. [3] | Taiwan | 5 | Male/unclear | CSF (-) |  | CSF (-) | N | N | Y | (+) |  |  |
| Tung Y R, et al. [3] | Taiwan | 9 | Male/unclear | CSF (-) |  | CSF (+) | Y | N | Y | (-) |  | Y |
| Tung Y R, et al. [3] | Taiwan | 11 | Male/unclear | CSF (-) |  | CSF (-) | N | N | Y | (+) |  |  |
| Tung Y R, et al. [3] | Taiwan | 5 | Male/unclear | CSF (-) |  | CSF (-) | N | N | Y | (+) |  |  |
| Geary S, et al. [4] | USA | 24 | Female | CSF (-) |  | CSF (+) | Y | N |  | (-); 11 |  | Y |
| Pike J, et al. [5] | Canada | 23 | Male | Cervical mass biopsy: necrotizing granuloma) |  | Gastric aspirate (+) | N | Y |  | (+) |  | Y |
| Uysal G, et al. [6] | Turkey | 4.5 | Male | Gastric aspirates (-) | Gastric aspirates (-) | Gastric acid aspirate (+) | N | Y | Y | (-) |  | Y |
| Decarie D, et al. [7] | Canada | 2 | Male |  |  | CSF (-); Gastric aspirate (-) | N | N |  | (+) |  | Y |
| Spyridis N, et al. [8] | Greece | 4 | Female | Skin lesions: (+) | CSF (+) |  | Y | Y | N | 16 |  | Y |
| Goić-Barišić I, et al. [9] | Croatia | 7 | Female |  |  | CSF (+) | Y | N | Y | (-); after 36 days (+) |  | Y |
| Nanda A, et al. [10] | Kuwait | 13 | Female |  |  | CSF (+) | Y | N |  | (-) |  | Unclear, AFB |
| Zorn-Olexa C, et al. [11] | France | 10 | Female | CSF (-) | CSF (-) | Gastric aspirate (+) | N | Y | Y | 17 |  | Y |
| Radmanesh F, et al. [12] | USA | 14 | Male | Peritoneal biopsy: granuloma formation with caseous necrosis. | CSF (+) | Tissue (-); Gastric washings (-) | Y | Y |  | (-) |  | Y |
| Katragkou A, et al. [13] | Greece | 18 | Male |  | CSF (+) | CSF (+) | Y | N | N | (-) | Indeterminate | Y |
| Smith B B, et al. [14] | Australia | 17 | Male |  | CSF (+); Endotracheal aspirate specimens (+) | CSF (+) | Y | Y | N | 5 | QFT(+) | Y |
| Smith B B, et al. | Australia | 18 | Male |  | CSF (+), BALF (+); Gastric aspirate (+) | CSF (+); Gastric aspirate (+); BAL fluid (+) | Y | Y | N | 12 |  | Y |
| Yilmaz R, et al. [15] | Turkey | 7 | Female | CSF (-) |  |  | N | N | Y | 1 |  |  |
| Palm S, et al. [16] | USA | 12 | Female |  |  | Gastric ﬂuid (+); CSF (+) | Y | Y |  | (+) |  | Y |
| Hoyos-Orrego Á, et al. [17] | Colombia | 1 | Male | Gastric aspirates (+); CSF (-) | CSF (+) | Gastric aspirates (+); CSF (-) | Y | Y |  | (-) |  | Y |
| Paul M, et al. [18] | USA | 8 | female | CSF (-); gastric lavage (-) | CSF (-) | CSF (+); Gastric lavage (+) | Y | Y |  | 10 |  | Y |
| Said M, et al. [19] | Australia | 11 | male | Endotracheal aspirate sample (+) |  | CSF (+) | Y | Y | N | (+) | QFT(+) | Y |
| Anandakrishnan P, et al. [20] | Malaysia | 8 | Female | CSF (-); Lymph nodes biopsy: (+) | Lymph nodes biopsy: Xpert (+) | Lymph nodes: (+) | N | Y | Y | (-) |  | Y |
| Ramírez M, et al. [21] | Colombia | 11 | MALE | CSF (-), Gastric aspirate (-); Brain biopsy：TB evidence and AFB (+) | CSF (-) | CSF (-); Gastric aspirate (-) | Y | N | Y | (-) |  | Unclear, AFB |
| Rosales Magallanes G. [22] | Mexico | 11 | male | CSF (-) | CSF (-) |  | N | N | N | (-) | QFT(+) |  |
| Huynh J, et al. [23] | Australia | 23 | Female |  | CSF (+); Gastric aspirates: Xpert Ultra (+) |  | Y | Y |  | 20 | IGRA (+) | Y |
| Andriescu E C, et al. [24] | USA | 10 | female | CSF (-) |  | CSF (-) | N | N | N | 15 | T-SPOT.TB(+) | Y |
| Foreman E, Raju S S. [25] | USA | 8 | Female | Sputum (+) |  |  | N | Y |  | (-);(+) |  | Unclear, AFB |
| Sánchez-Códez M I, et al. [26] | Spain | 10 | Female |  | CSF (+); Gastric aspirates: Xpert (+) |  | Y | Y | N | 0 | IGRA(+) | Y |
| Furuichi M, et al. [27] | Japan | 16 | male | Brain tissue: non-caseous granulomatous inflammation with no evidence of malignancy or infection. | CSF (-); CSF (+); ventricular catheters and brain tissues (+) | CSF (-) | Y | N | Y |  | IGRA(-) | BCG |
| Yang J H, et al. [28] | USA | 7 | Female | CSF(-); Mediastinal mass: AFB-positive necrotizing granulomas | CSF (-) | CSF (-); Mediastinal mass (+) | N | Y | N | 10 | QFT(+) | Y |
| Kondo S, et al. [29] | Japan | 0 | Male |  |  |  | N | N | N | 0 |  |  |
| Kondo S, et al. [29] | Japan | 0 | Male |  |  |  | N | N | N | 10 |  |  |
| Kondo S, et al. [29] | Japan | 0 | Male |  |  |  | N | N | N | 0 |  |  |
| Kondo S, et al. [30] | Japan | 12 | Male | CSF (-); Gastric ﬂuid (-) |  | CSF (+); Gastric ﬂuid (+) | Y | Y | N | 13 |  |  |
| Kondo S, et al. [31] | Japan | 4 | Female | Gastric ﬂuid (-); CSF (-) |  | Gastric ﬂuid (+); CSF (+) | Y | Y | N | 0 |  | Y |
| Kondo S, et al. [31] | Japan | 4 | Male | Gastric ﬂuid (+); CSF (-) | Gastric ﬂuid (-); CSF (-) | Gastric ﬂuid (+); CSF (-) | N | Y | N | 0 |  | Y |
| Kondo S, et al. [31] | Japan | 6 | Male | Gastric ﬂuid (+); CSF (+) | Gastric ﬂuid (+); CSF (+) | Gastric ﬂuid (+); CSF (+) | Y | Y | N | 10 |  | Y |
| Kondo S, et al. [31] | Japan | 7 | Male | Gastric ﬂuid (+); CSF (+) | Gastric ﬂuid (+); CSF (+) | Gastric ﬂuid (+); CSF (+) | Y | Y | Y | 0 |  | Y |
| Kondo S, et al. [31] | Japan | 8 | Male | Gastric ﬂuid (-); CSF (-) |  | Gastric ﬂuid (+); CSF (-) | N | Y | N | 0 |  | Y |
| Kondo S, et al. [31] | Japan | 11 | Female | Gastric ﬂuid (-); CSF (-) |  | Gastric ﬂuid (-); CSF (+) | Y | N | N | 9 |  | Y |
| Kondo S, et al. [31] | Japan | 11 | Male | Gastric ﬂuid (-); CSF (-) | Gastric ﬂuid (-); CSF (+) | Gastric ﬂuid (+); CSF (+) | Y | Y | Y | 5 |  | Y |
| Kondo S, et al. [31] | Japan | 11 | Male | Gastric ﬂuid (-); CSF (-) | Gastric ﬂuid (-); CSF (-) | Gastric ﬂuid (+); CSF (+) | Y | Y | N | 13 |  | Y |
| Kondo S, et al. [31] | Japan | 16 | Female | Gastric ﬂuid (+); CSF (+) | Gastric ﬂuid (+); CSF (+) | Gastric ﬂuid (+); CSF (+) | Y | Y | N | 11 |  | Y |

**References**

1. Janner D, Kirk S, McLeary M. Cerebral tuberculosis without neurologic signs and with normal cerebrospinal fluid. Pediatr Infect Dis J. 2000;19(8):763-4. doi:10.1097/00006454-200008000-00021.

2. Pejham S, Altman R, Li KI, Munoz JL. Congenital tuberculosis with facial nerve palsy. Pediatr Infect Dis J. 2002;21(11):1085-6. doi:10.1097/00006454-200211000-00027.

3. Tung Y-R, Lai M-C, Lui C-C, Tsai K-L, Huang L-T, Chang Y-C, et al. Tuberculous meningitis in infancy. Pediatric Neurology. 2002;27(4):262-6. doi:10.1016/S0887-8994(02)00431-9.

4. Geary S, Agnew M. 2-year-old with tuberculosis meningitis: a case study. Journal of Neuroscience Nursing. 2004;36(2):90-4. doi:10.1097/01376517-200404000-00006.

5. Pike J, Steinbok P, Reilly CW. Cervical intramedullary tuberculoma and tuberculous kyphosis in a 23-month-old child: case report. Canadian Journal of Surgery. 2005;48(3):247-50.

6. Uysal G, Gursoy T, Altunc U, Guven A. Asymptomatic pons tuberculoma in an infant with miliary tuberculosis. Neurosciences (Riyadh). 2005;10(4):309-11.

7. Décarie D, Grenier JL, Allard A. Outbreak of tuberculosis in the Laurentian region, 2005. Can Commun Dis Rep. 2006;32(19):226-9.

8. Spyridis N, Georgouli H, Tsoukatou T, Sakou I, Tsolia M, Miriokefalitakis N, et al. Severe disseminated tuberculosis in a 4-month-old infant initially presenting with multiform cutaneous lesions. Scand J Infect Dis. 2006;38(4):306-8. doi:10.1080/00365540500361310.

9. Pulmonary tuberculosis with meningitis in a 7-month-old infant. Thieme Medical Publishing Inc.; 2007. p. 51-4.

10. Nanda A, Nanda M, Dvorak R, Al-sabah H, Alsaleh QA. Bullous pemphigoid (BP) in an infant complicated by tuberculous meningoencephalitis. International Journal of Dermatology. 2007;46(9):964-6. doi:10.1111/j.1365-4632.2007.03194.x.

11. Zorn-Olexa C, Laugel V, Martin AdS, Donato L, Fischbach M. Multiple intracranial tuberculomas associated with partial status epilepticus and refractory infantile spasms. Journal of Child Neurology. 2008;23(4):459-62. doi:10.1177/0883073807309252.

12. Radmanesh F, Nejat F, El Khashab M. Cerebral infarction as the first presentation of tuberculosis in an infant: a case report. J Microbiol Immunol Infect. 2010;43(3):249-52. doi:10.1016/s1684-1182(10)60039-4.

13. Katragkou A, Antachopoulos C, Hatziagorou E, Sdougka M, Roilides E, Tsanakas J. Drug-resistant tuberculosis in two children in Greece: Report of the first extensively drug-resistant case. European Journal of Pediatrics. 2013;172(4):563-7. doi:10.1007/s00431-012-1811-8.

14. Smith BB, Hazelton BJ, Heywood AE, Snelling TL, Peacock KM, Macartney KK. Disseminated tuberculosis and tuberculous meningitis in Australian-born children; case reports and review of current epidemiology and management. J Paediatr Child Health. 2013;49(3):E246-50. doi:10.1111/jpc.12035.

15. Yilmaz R, Kundak AA, Sezer T, Özer S, Esmeray H, Kazanci NO. Idiopathic infantile hypercalcemia or an extrapulmonary complication of tuberculosis? Tuberkuloz ve Toraks. 2013;61(1):43-6. doi:10.5578/tt.536.

16. Palm S, Balan A, Ramji F. Persistent fever in an infant with meningitis. Journal of Investigative Medicine. 2014;62(2):471. doi:10.231/JIM.0000000000000055.

17. Hoyos-Orrego Á, Trujillo-Honeysberg M, Diazgranados-Cuenca L. Congenital Tuberculosis as a Result of Disseminated Maternal Disease: Case Report. Tuberc Respir Dis (Seoul). 2015;78(4):450-4. doi:10.4046/trd.2015.78.4.450.

18. Paul M, Shehab K, Nguyen T. Case 1: Progressive Hypotonia and Decreased Alertness in an 8-month-old Girl. Pediatr Rev. 2017;38(8):383. doi:10.1542/pir.2014-0137.

19. Said M, Uppal P, Bye A, Palasanthiran P. Unusual case of tuberculous meningitis with discordant ventricular and lumbar cerebrospinal fluid; lessons in the era of world-wide migration. J Paediatr Child Health. 2018;54(1):93-5. doi:10.1111/jpc.13665.

20. Anandakrishnan P, Khoo TB. Unusual case of cerebral demyelination and bilateral optic neuritis in an infant with suppurative BCG lymphadenitis. BMJ Case Rep. 2018;2018. doi:10.1136/bcr-2018-224496.

21. Ramírez M, Cortés E, Betancur J, Garcés C. [Cerebral tuberculosis without meningitis in a immunocompetent child]. Rev Chilena Infectol. 2018;35(2):207-12. doi:10.4067/s0716-10182018000200207.

22. Francisco Rosales-Magallanes G. Meningitis tuberculosa en un lactante, a propósito de la aplicación de BCG a migrantes de zonas endémicas. Revista Mexicana de Pediatria. 2018;85(6):222-5.

23. Huynh J, Vosu J, Marais BJ, Britton PN. Multidrug-resistant tuberculous meningitis in a returned traveller. J Paediatr Child Health. 2019;55(8):981-4. doi:10.1111/jpc.14387.

24. Andriescu EC, Khetan NG, Mazur L, Smith KC. Tuberculosis Meningitis in a 10-Month-Old Living in an Immigrant Township. Clin Pediatr (Phila). 2019;58(11-12):1341-4. doi:10.1177/0009922819852997.

25. Foreman E, Surapa Raju S. Communicating hydrocephalus in an infant with tuberculosis meningitis. Journal of Investigative Medicine. 2019;67(2):483. doi:10.1136/jim-2018-000974.335.

26. Sánchez-Códez MI, Lubián-Gutiérrez M, Fernández-Bravo C, Ley-Martos M. Pediatric miliary tuberculosis presenting with stroke: contribution to the paper "Tuberculosis of the central nervous system in children". 2019. p. 1273-5.

27. Furuichi M, Mori F, Uejima Y, Sato S, Kurihara J, Kawano Y, et al. A case of Mycobacterium bovis Bacillus Calmette-Guérin (BCG) strain meningitis and ventriculitis following BCG vaccination. Int J Infect Dis. 2020;100:373-6. doi:10.1016/j.ijid.2020.09.1419.

28. Yang JH, Vuong KT, Moodley A, Chuang NA, Chen DY. A Case of Tuberculosis-Associated Acute Disseminated Encephalomyelitis in a Seven-Month-Old Infant. Cureus. 2021;13(7):e16299. doi:10.7759/cureus.16299.

29. Kondo S, Ito M. [Infants and children who developed tuberculosis following contacts investigation]. Kekkaku. 2003;78(12):747-50.

30. Kondo S, Miyagawa T. Three cases of tuberculosis children who demonstrated paradoxical worsening during different stage of treatment. Kekkaku : [Tuberculosis]. 2006;81(5):375-9.

31. Kondo S, Ito M. [Usefulness of cranial and chest imaging in the diagnosis of tuberculous meningitis among infants and young children]. Kekkaku. 2003;78(2):89-93.
